# Enhancement of Photostabilization of Poly(Vinyl Chloride) in the Presence of Tin–Cephalexin Complexes

**DOI:** 10.3390/polym15030550

**Published:** 2023-01-20

**Authors:** Rafid R. Arraq, Angham G. Hadi, Dina S. Ahmed, Gamal A. El-Hiti, Benson M. Kariuki, Amani A. Husain, Muna Bufaroosha, Emad Yousif

**Affiliations:** 1 Department of Chemistry, College of Science, Babylon University, Babil 51002, Iraq; 2 Department of Medical Instrumentation Engineering, Al-Mansour University College, Baghdad 64201, Iraq; 3 Department of Optometry, College of Applied Medical Sciences, King Saud University, Riyadh 11433, Saudi Arabia; 4 School of Chemistry, Cardiff University, Main Building, Park Place, Cardiff CF10 3AT, UK; 5 Polymer Research Unit, College of Science, Al-Mustansiriyah University, Baghdad 10052, Iraq; 6 Department of Chemistry, College of Science, United Arab Emirates University, P.O. Box 15551, Al-Ain 1818, United Arab Emirates; 7 Department of Chemistry, College of Science, Al-Nahrain University, Baghdad 64021, Iraq

**Keywords:** tin–cephalexin complexes, poly(vinyl chloride), photodegradation, photostability, weight loss, surface morphology

## Abstract

Poly(vinyl chloride), PVC, has many attractive properties, including low cost of manufacture, resistance to acid and alkali corrosion, and ease of molding. However, PVC suffers from aging in harsh conditions, leading to the shortening of its useful life. Stability to irradiation, for example, can be improved through the incorporation of additives to PVC. The design, synthesis, and application of new stabilizers continue to attract attention. The current work investigates the effect of three tin–cephalexin complexes on the stability of PVC on irradiation with ultraviolet (UV) light (λ = 313 nm) at 25 °C for a long duration. The PVC was blended with tin–cephalexin complexes at low concentrations (0.5% by weight), and thin films (around 40 µm) were made from the mixed materials. Various methods, including weight loss, infrared spectroscopy, and surface inspection of irradiated films were used to investigate the role played by these additives in the inhibition of PVC photodecomposition. The results confirmed that the additives led to a significant reduction in the rate of photodecomposition of the PVC blends. Tin–cephalexin complexes can absorb harmful radiation, deactivate hydrogen chloride, and scavenge high-energy species such as peroxides, therefore acting as stabilizers for PVC.

## 1. Introduction

The consumption of plastics has increased progressively due to their numerous applications [1,2]. Poly(vinyl chloride), PVC, is widely used due to its many attractive properties, including low production cost; manufacture in different shapes and forms; and resistance to flame, acid, and alkali corrosion. PVC is used in construction materials, flooring, packing, cables, wires, pipes, foams, and fibers [3,4,5]. A drawback in the use of PVC is that environmental factors, such as high temperature, light, and humidity, combined with the presence of oxygenated species, can lead to undesirable chemical and physical changes in the polymer chains [6]. This process of photodegradation results in the formation of reactive species such as hydrogen, chloride, alcohol, acid, peroxide, and hydroperoxide free radicals that initiate PVC autocatalytic degradation. The degradation initially takes place at sites of defects and abnormalities and leads to C−C bond cleavage, cross-linking, and chain scission producing unsaturated residues and PVC chains containing carbonyl groups, for example [7]. PVC photodegradation results in color change, deformation, cracking, the appearance of spots, and weight loss due to the elimination of small fragments including volatiles (e.g., hydrogen chloride, HCl) [8,9,10,11,12]. The inclusion of additives during the manufacture of PVC is one way of elongating its useful life [13,14,15].

The PVC additives should ideally be easy to synthesize, cheap to produce, highly stable, non-volatile, non-toxic, and effective in low concentrations. They should be capable of adsorbing HCl produced during PVC photodecomposition or suppressing its formation and therefore suppressing the formation of polyene residues within the chains. In addition, PVC additives should be able to absorb UV irradiation directly and resist discoloration [16,17,18,19]. Additives containing metals (e.g., zinc), nanomaterials (e.g., titanium dioxides), tetrachlorobiphenyl, *tris*(di-*tert*-butylphenyl)phosphite, and *bis*(2-ethylhexyl)phthalate have been shown to act as PVC plasticizers [20,21,22,23,24,25]. These additives act as common and efficient HCl plasticizers and absorbers. However, many of the additives are toxic and considered hazardous for humans, animals, and the environment and are therefore banned from use [26,27,28,29]. Attention has therefore continued to be paid to the design and synthesis of other PVC additives. Examples include highly aromatic polyphosphates, Schiff bases, and heterocycles that can act as effective PVC photostabilizers [30,31,32,33,34].

Organic compounds containing tin are involved in many applications [35,36,37,38,39]. They act as wood preservatives, disinfectants, catalysts, agrochemicals, biocides, and polymer stabilizers [40]. The design and synthesis of new organic compounds containing tin are still of interest to many researchers. In continuation of our research into alternative photostabilizers for polymers, we now report the use of three tin–cephalexin complexes for the inhibition of photodegradation in PVC. Cephalexin (a beta-lactam) is used as an antibiotic to treat skin, bone, ear, and urinary tract bacterial infections [41,42]. It is a stable molecule and contains aromatic moieties (phenyl and heterocycles) and high content of heteroatoms (39.8%; S, O, and N). Therefore, cephalexin has the qualities necessary to inhibit PVC photodecomposition in complexation with tin.

## 2. Materials and Methods

### 2.1. General

Chemicals, solvents, and reagents were sourced from Merck (Gillingham, UK). PVC with an average molecular weight of around 180,000 g/mol was provided by Petkim Petrokimya (Istanbul, Turkey). For recording the FTIR spectra, a Shimadzu FTIR-8300 spectrophotometer (Tokyo, Japan) was used. UV light with a maximum wavelength (λ_max_) of 365 nm and a light intensity of 6.2 × 10^–9^ Einstein dm^–3^ s^–1^ was used to irradiate the samples at 25 °C using a Q-Panel tester (Homestead, FL, USA). The tester has two UV fluorescent lamps (λ_max_ = 365 nm, 40 watts), one on each side. The films were placed vertically 10 cm away from the source of the irradiation light and parallel to the UV lamps. The films were rotated occasionally to enable even irradiation from all sides. A Meiji Techno Microscope (Tokyo, Japan), an FEI Inspect S50 microscope (Czechia, Czech Republic), and a Veeco microscope (Plainview, NY, USA) were used to inspect the surface of the PVC films.

### 2.2. Synthesis of Complexes **1**–**3**

**Scheme 1:**
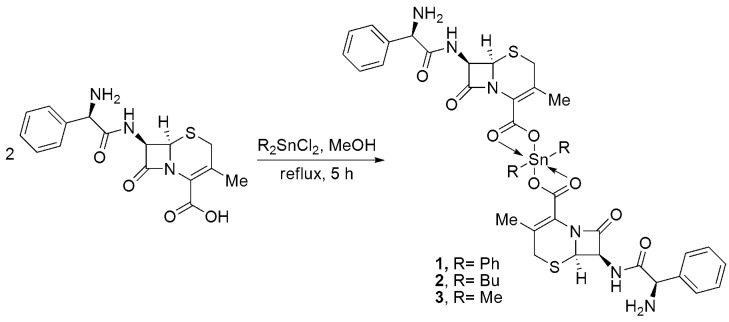
Synthesis of tin–cephalexin complexes **1**–**3**.

Tin–cephalexin complexes **1–3** (Scheme 1) were synthesized in high yields (78−98%) employing a reported procedure [43]. Reactions of excess cephalexin (2 mole equivalent) and disubstituted tin dichloride (diphenyltin dichloride, dibutyltin dichloride, and dimethyltin dichloride) in methanol (MeOH) under reflux for 5 h gave **1**–**3**. The spectroscopic and analytical data of **1**−**3** agreed with those reported [43].

### 2.3. PVC Films Preparation

PVC (5 g) and 25 mg of the appropriate complex **1**, **2**, or **3** were mixed in tetrahydrofuran (THF; 100 mL). The mixture was stirred at 25 °C for 2 h, and the homogenous solution obtained was poured into a plate with holes (15; thickness = 40 µm). The plate was kept for 24 h at 25 °C to remove the THF. The resultant films produced were then dried in a vacuum oven (40 °C; 8 h) for complete elimination of any residual THF.

### 2.4. IR Spectroscopy of PVC

Photodegradation of PVC produces polymeric fragments with carbonyl (C=O) and polyene (C=C) groups due to the elimination of HCl from the polymer chains [44,45]. Thus, FTIR spectroscopy enabled the detection of the changes in the intensity of C=O (1714 cm^–1^) and C=C (1618 cm^–1^) absorption bands as photodegradation progressed. The growth in the peak intensities was compared with that for the C–H bonds (1328 cm^–1^), which does not change significantly during PVC irradiation. Functional group indexes (I_s_) for both the C=O (I_C=O_) and the C=C (I_C=C_) groups were calculated using Equation (1) from the absorbance of the functional group (A_s_) and that of the reference band (A_r_) [46].

(1) $$I_{s}=\frac{A_{s}}{A_{r}}$$

### 2.5. Weight Loss of PVC

Elimination of small polymeric residues and volatiles during PVC photodegradation leads to weight loss. Equation (2) was used to calculate the PVC weight loss percentage from W_0_ (weight of nonirradiated film) and W_t_ (weight of irradiated films) [47].

(2) $$Weight\;loss\left(\%\right)=\frac{W_{0}-W_{t}}{W_{0}}\times 100$$

## 3. Results and Discussion

### 3.1. IR Spectroscopy of PVC

The photodegradation process of PVC is initiated when the polymeric materials are exposed to UV light for an extended period in an environment containing oxygen. This process produces reactive species (free radicals) that cause cross-linking of PVC as well as small fragments. The polymeric residues most eliminated contain C=O (ketones, chloroketones, chlorocarboxylic acid, and acid chloride) and C=C (polyene) [48,49]. The mechanism of PVC photodegradation is a complex chain dehydrochlorination. The photodegradation of PVC leads to the formation of polymeric fragments that contain conjugated doubles due to the elimination of HCl. The process is a chain reaction that involves zipper elimination to form conjugated polyenes that contain up to 25 double bonds [50].

**Figure 1:**
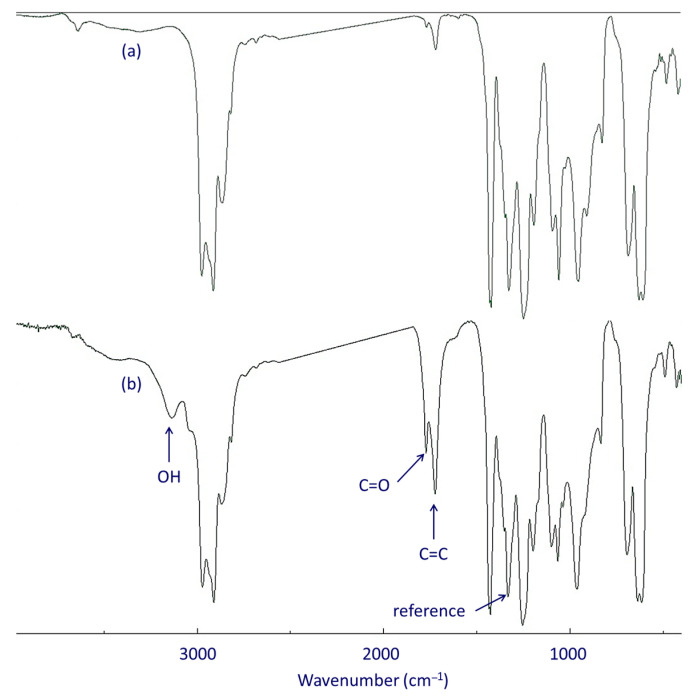
FTIR spectra of the unblended PVC film (**a**) before irradiation and (**b**) after 300 h of irradiation.

The blends of PVC and tin–cephalexin complexes **1**–**3** were irradiated for durations up to 300 h, followed by recording the IR spectra. The changes in the absorption bands for C=O (1732 cm^–1^) and C=C (1606 cm^–1^) were compared to that for the C–H bond (1328 cm^–1^). As Figure 1 shows, the signals for the C=O and C=C groups for the blank PVC film grew significantly during irradiation compared with the non-irradiated film.

**Figure 2:**
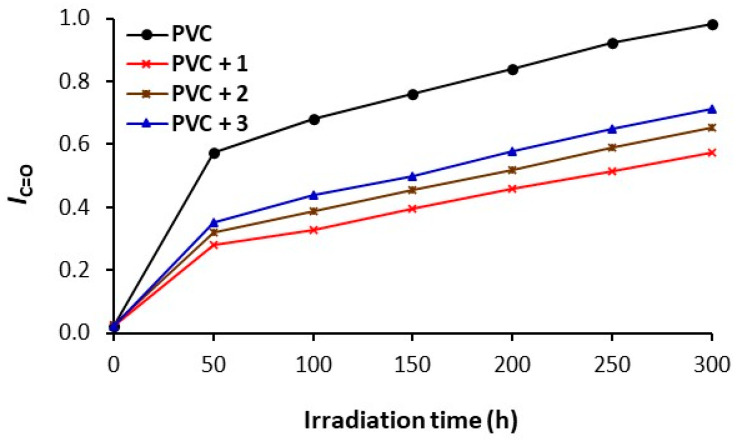
Effect of the time of irradiation on the I_C=O_ of PVC films.

**Figure 3:**
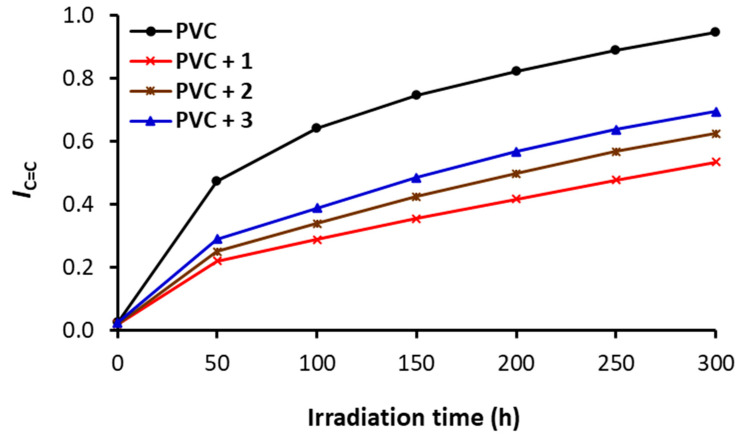
Effect of the time of irradiation on the I_C=C_ of PVC films.

The carbonyl (I_C=O_) and alkene (I_C=C_) indices for the films irradiated for different periods were calculated using Equation (1) and are represented graphically in Figure 2 and Figure 3. It is evident that tin–cephalexin complexes **1**–**3** stabilized the PVC substantially since both carbonyl and alkene indices were much higher for the unblended film compared with those containing additives. Tin complex **1**, which contains aromatic substituents (phenyl groups), was a more efficient PVC photostabilizer compared to those with aliphatic substituents (butyl and methyl groups). At the end of the irradiation process, for example, the I_C=O_ was 0.98 for the unblended PVC film and 0.58, 0.65, and 0.71 for the blends with complexes **1**, **2**, and **3**, respectively. Similarly, I_C=C_ for the irradiated materials (300 h) was 0.95 for the unblended PVC film and 0.54, 0.63, and 0.70 for the blends containing complexes **1**, **2**, and **3**, respectively.

### 3.2. Weight Loss of PVC

**Figure 4:**
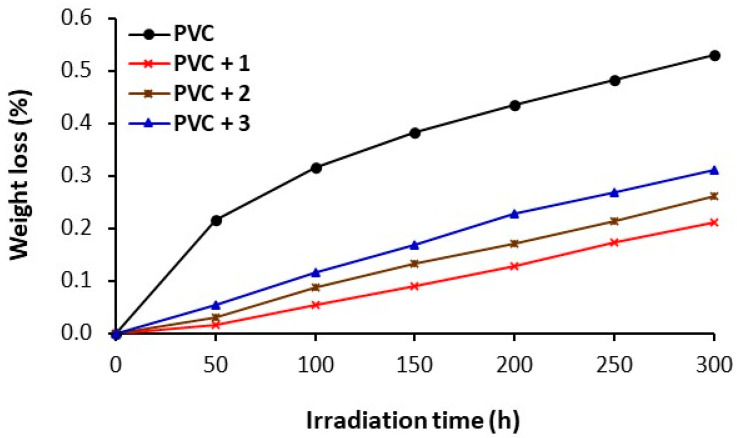
Effect of the time of irradiation on the weight of PVC films.

The release of HCl from PVC chains at high temperatures causes discoloration. In addition, it leads to the ejection of small fragments with loss in mass [51,52]. The PVC weight loss during UV irradiation was explored. The films were irradiated (50–300 h) and the percentage loss in PVC weight was calculated using Equation (2). Figure 4 shows that the longer the irradiation time, the greater the weight loss. Additionally, the presence of tin–cephalexin complexes **1**–**3** reduced PVC weight loss. After 300 h of irradiation, the weight loss percentage was 0.53% for the unblended PVC film and 0.21, 0.26, and 0.31% for the blends containing complexes **1**, **2**, and **3**, respectively. The results shown in Figure 2, Figure 3 and Figure 4 were consistent and indicated that the order of photostabilization efficiency of the metal complexes as PVC additives was **1**, **2**, and **3**.

### 3.3. Surface Morphology of PVC

**Figure 5:**
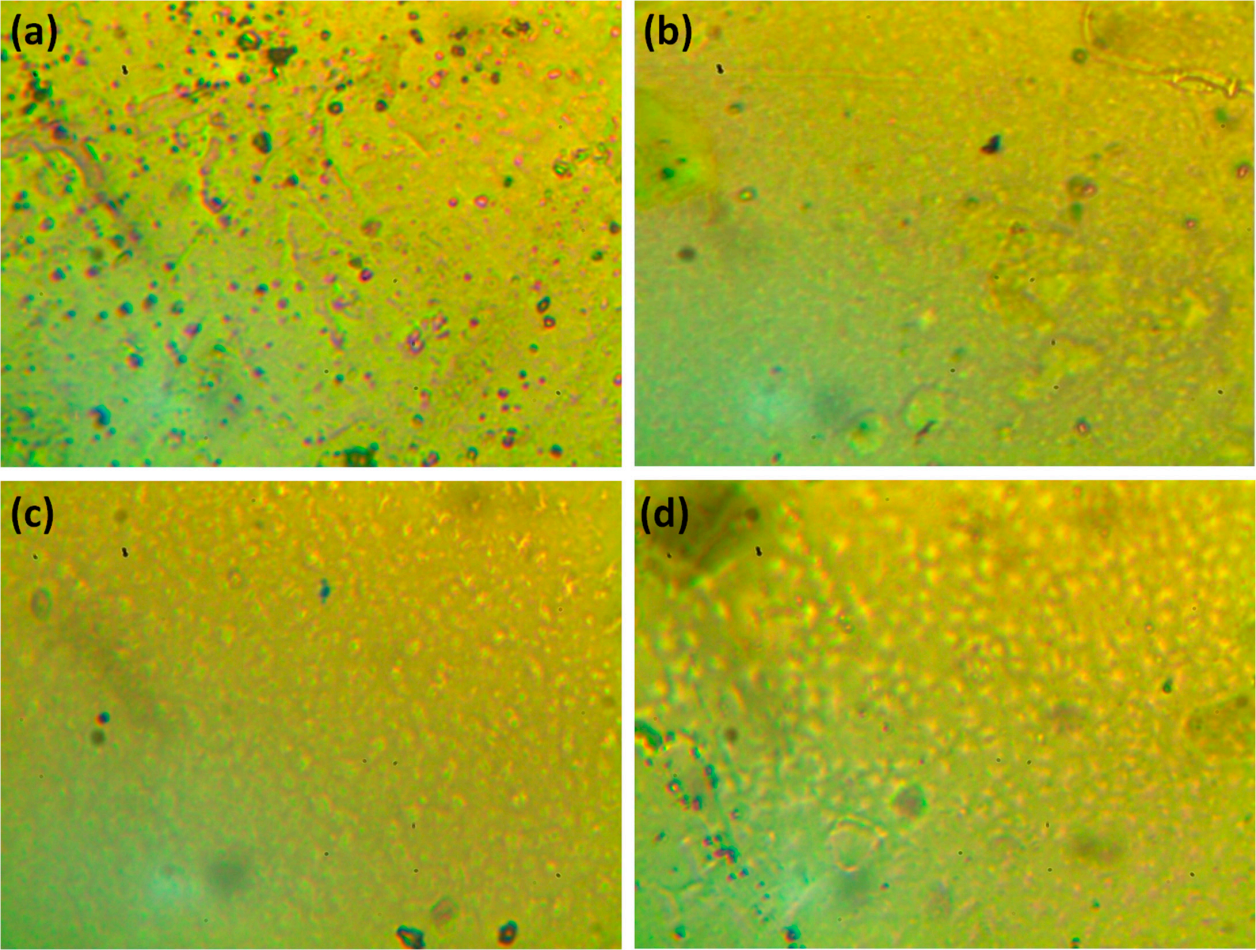
Microscope images (400× magnifications) of irradiated (**a**) pure PVC, (**b**) PVC + **1**, (**c**) PVC + **2**, and (**d**) PVC + **3**.

Inspection of the irradiated PVC film surface microscopically provides information about irregularity, roughness, and crystallinity. Another indication of damage (e.g., the appearance of spots, cracks, and darkness) to the surface of the irradiated materials is also provided [53,54,55,56]. The optical microscopy images of the PVC films indicated a tendency to form a rough, irregular, and heterogeneous surface after irradiation (Figure 5). Additionally, the presence of spots and cracks as well as dark, irregular, and rough areas was revealed. The surface damage and irregularities were more significant in the case of the unblended PVC, indicating the important role played by the tin–cephalexin complexes **1**–**3** in the stabilization of the polymeric materials. The least noticeable damage was observed on the surface of the PVC film blended with complex **1**.

**Figure 6:**
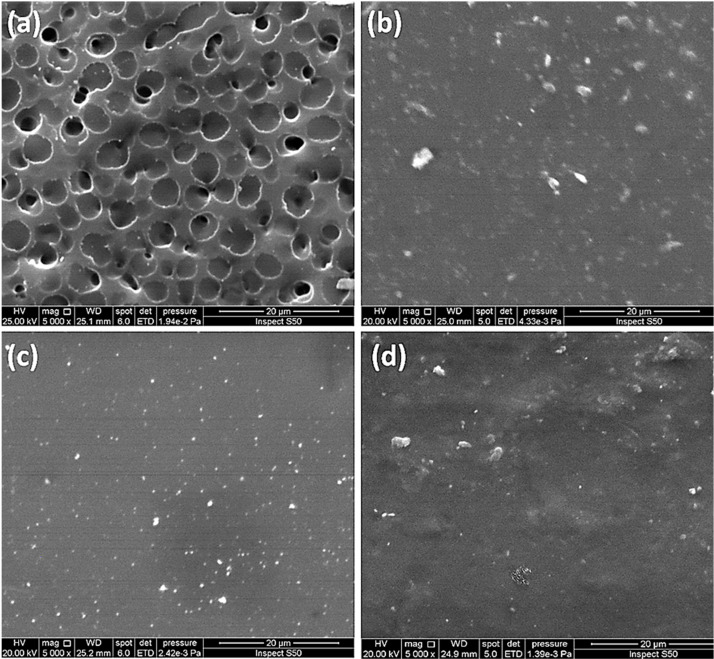
SEM images of irradiated (**a**) pure PVC, (**b**) PVC + **1**, (**c**) PVC + **2**, and (**d**) PVC + **3**.

The scanning electron microscopy (SEM) technique provides high-resolution clear undistorted images of the PVC surface. The images can provide information about homogeneity and irregularity in the surface of the materials [54]. The surface damage was very significant in the case of the unblended PVC relative to those of the blends containing tin–cephalexin complexes **1**–**3** (Figure 6). The surface of the unblended PVC showed many holes and groves that had formed due to the high rate of HCl elimination on irradiation [57].

**Figure 7:**
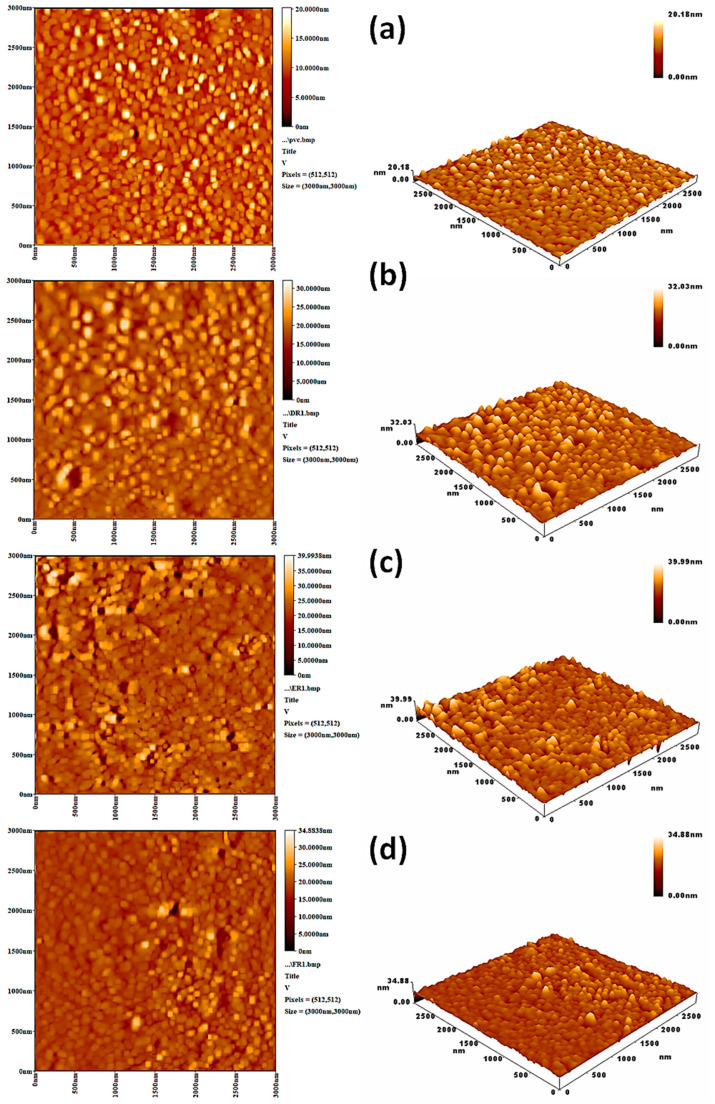
AFM images of irradiated (**a**) pure PVC, (**b**) PVC + **1**, (**c**) PVC + **2**, and (**d**) PVC + **3**.

The atomic force microscopy (AFM) imaging was also used to record two- and three-dimensional images of the films (Figure 7). The images indicated that the surface roughness of the unblended PVC film was higher than those of the blends containing tin–cephalexin complexes **1**–**3**. At the end of the irradiation process, the roughness factors (Rq) were 516.2 for the unblended film and 38.2, 41.2, and 46.3 for the PVC film blends containing tin–cephalexin complexes **1**, **2**, and **3**, respectively. Notably, the use of complex **1** led to a 13.5-fold reduction in the Rq, which is higher than observed (reduction in the Rq by fold = 5.2–12.9) for other tin complexes comprising different aromatic moieties [58,59,60,61]. However, additives containing high contents of heteroatoms and aromatic residues were more efficient (reduction in the Rq by fold = 15.4–21.2) in reducing the PVC surface roughness than the current ones [62,63,64,65,66].

### 3.4. Suggested Mechanisms for PVC Photostabilization

The effectiveness of tin–cephalexin complexes **1**–**3** as PVC photostabilizers are in the order **1** > **2** > **3**. Complexes **1**–**3** can reduce PVC photodegradation through different processes. Complex **1** contains extra phenyl groups (aromatic), while **2** and **3** contain butyl and methyl ligands (aliphatic) suggesting that the degree of aromaticity of additives is important in stabilizing PVC. It is possible that the aromatic residues (phenyl and heterocycles) within the complexes absorb UV irradiation directly [67,68]. The absorbed irradiation could be released as heat that does not harm the PVC chains. In addition, the polarity of the bonds linked to heteroatoms within complexes could help the coordination with the polarized C–Cl bonds in PVC. Such coordination eases the transfer of energy from the excited state complexes and PVC, enabling a slow dissipation of energy.

**Scheme 2:**
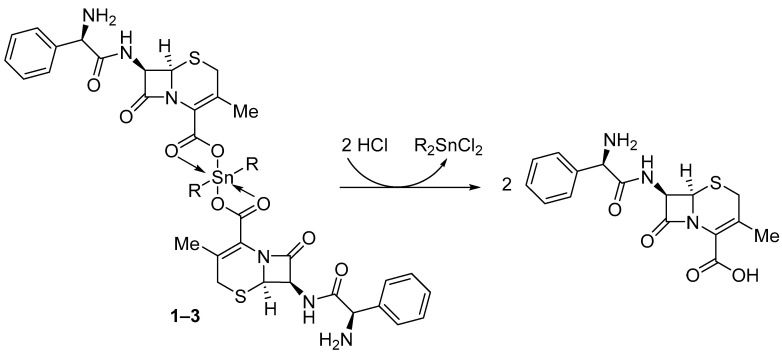
Cephalexin–tin complexes act as HCl scavengers.

Complexes **1**–**3** contain a tin atom that is a Lewis acid and, therefore, acts as an HCl scavenger (Scheme 2). The reaction between complexes **1**–**3** and HCl produced from PVC during the irradiation process leads to the formation of disubstituted tin dichloride and the release of the cephalexin ligand.

**Scheme 3:**
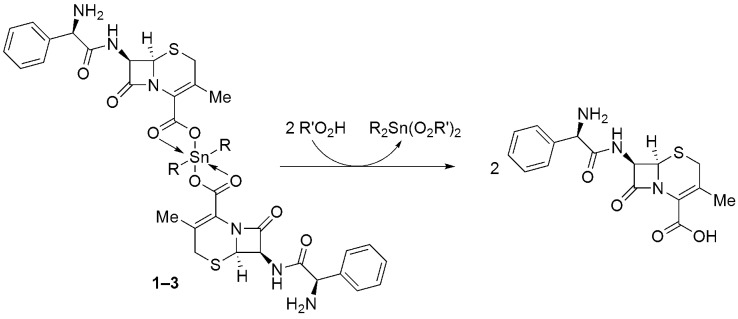
Cephalexin–tin complexes act as peroxide decomposers.

Finally, tin–cephalexin complexes **1**–**3** can act as free radical decomposers. They deactivate the harmful and reactive species such as peroxides that are produced in the process of PVC photodegradation. For example, the complexes decompose hydroperoxides (R’O_2_H) produced due to the irradiation of PVC and release peroxide-containing tin and the ligand (Scheme 3). In addition, the complexes interact with peroxides and form intermediates capable of stabilization by the resonance of phenyl groups, for example [69]. 

## 4. Conclusions

Three tin complexes with the cephalexin moiety were synthesized and assessed as PVC additives. The additives were used in low proportions and were demonstrated to significantly reduce the photodegradation of PVC. The formation of residues containing functional groups, weight loss, and deformation of the surface were much lower in the presence of additives in comparison to the pure film. The tin–cephalexin complexes act as scavengers for active species such as peroxides, volatiles such as hydrogen chloride, quenchers for energy, and absorbers of harmful irradiation. The additive containing a higher proportion of aromatic substituents (phenyl groups) showed better performance than those containing aliphatic residues (butyl and methyl groups). The aromatic moieties possibly neutralize the reactive intermediates produced by irradiation through resonance stabilization acting as UV absorbers. Future research should pay attention to the hazardous effect that might be associated with the use of metal complexes as PVC additives and the possible leakage of metals into the environment.

## Data Availability

Data are contained within the article.
